# Assessing the Impact of Internet Skills on Depressive Symptoms Among Chinese Middle-Aged and Older Adults: Cross-Sectional Instrumental Variables Analysis

**DOI:** 10.2196/50880

**Published:** 2024-03-21

**Authors:** Aruhan Mu, Zhiyong Liu

**Affiliations:** 1 School of Medicine and Health Management, Huazhong University of Science and Technology, Wuhan, China

**Keywords:** internet skills, depression, second-level digital divide, instrumental variables

## Abstract

**Background:**

The potential benefits of IT for the well-being of older adults have been widely anticipated. However, findings regarding the impact of internet use on depressive symptoms are inconsistent. As a result of IT’s exponential growth, internet skills have supplanted internet access as the source of the digital divide.

**Objective:**

This study evaluates the effect of internet skills on depressive symptoms through an instrumental variables (IV) approach.

**Methods:**

Data from the China Health and Retirement Longitudinal Study’s wave 4 (2018) were used. This included 16,949 community residents aged 45 years and older. To overcome the endogeneity issue, we used an IV approach.

**Results:**

Our results reveal the emergence of a second-level digital divide, the disparity in internet skills, among Chinese middle-aged and older adults. Liner regression suggests that a 1% increase in internet skills is associated with a 0.037% decrease in depressive symptoms (β=−.037, SE 0.009), which underestimates the causal effect. As expected, internet skills are an endogenous variable (*F* test *P* value <.001). IV regressions indicate that a 1% increase in internet skills reduces 1.135% (SE 0.471) to 1.741% (SE 0.297) of depressive symptoms. These 2 IV are neither weak (*F_–_*_1_=16.7 and 28.5; both >10) nor endogenous (Wu-Hausman test *P* value of .10; >.05 or >.01).

**Conclusions:**

Better mental health is predicted through improved and higher internet skills. Consequently, residents and policy makers in China should focus on bridging the digital divide in internet skills among middle-aged and older adults.

## Introduction

### Background

By 2050, there will be 2.1 billion individuals aged 60 years or older, with 80% of them living in low- and middle-income countries [1]. Late-life depression is a major public health challenge for this population because of its high prevalence and poor outcomes [2,3]. China, one of the low- and middle-income countries, is predicted to reach nearly 400 million older adults by 2050 [4]. Approximately 20%-31% of individuals aged 45 years or older in China had depression in 2015, with the highest risk among all age groups [5,6]. In 2013, the annual cost for individuals with mental disorders in China was US $3665, with depression and depressive symptoms accounting for 54% of the total [7,8]. With a rapidly aging population, late-life depression may damage the well-being of older adults; it also brings more burden to the family as well as the community. China accounts for nearly 17% of the global mental health burden [9]. In contrast, China’s mental health system has only 8.75 mental health workers per 100,000 residents [10], which calls for novel approaches with low cost and wide access to reduce depressive symptoms among middle-aged and older adults.

Given that the internet can improve one’s mental health by reducing social isolation and loneliness, it has attracted marked attention among researchers [11]. Prior work has investigated the correlation between depression symptoms and the frequency, type, and purpose of internet use, and identified potential mechanisms from the perspective of social connectedness [12-16]. Overall, these studies suggest that internet use among older adults offers new opportunities to prevent, support, and treat late-life depression in family and community contexts.

We aim to complement this research topic in 2 ways. First, previous studies have mainly measured internet use from the perspective of the first-level digital divide, that is, whether respondents have access to the internet [12,16,17]. With the rapid penetration of IT, internet skills (the capability of internet use) have replaced internet access as the most essential variable in characterizing IT use [18-20]. We believe that we have now entered the second level of the digital divide and thus chose internet skills to measure internet use. Second, the existing literature has derived results mainly from correlation analysis and yielded mixed evidence [13,16,21,22]. However, the findings of correlation analysis cannot provide interventional insights; only causal findings can do so [23]. Several modern economic approaches have been used in empirical studies to estimate the causal relation, such as the instrumental variables (IV) approach. Joshua D Angrist, who shares the 2021 Nobel Prize for methodological contributions to the analysis of causal relationships, adopted and developed the IV approach to quantify the impact of educational attainment on wage growth [24]. In the face of rising public health challenges in an aging society, policy makers and the public need robust insights. Therefore, our goal is to uncover the causal effects of internet skills on depressive symptoms in middle-aged and older adults and provide practical implications for promoting mental health through daily IT use.

Overall, IT-based mental health promotion programs could be an essential means to address the high prevalence of depression in older adults when they lack specialized medical resources [25]. As the internet spreads to older adults, we should pay more attention to their differences in internet skills rather than in internet access [20,26]. We should also focus on causal effects to provide more valuable evidence for public health practices [23]. Therefore, this study examines the causal effect of internet skills on depressive symptoms among middle-aged and older Chinese adults by using data from the 2018 China Health and Retirement Longitudinal Study (CHARLS). We used the IV approach to address the endogeneity issue and guide daily internet use practices among middle-aged and older adults to improve their mental health.

### Literature Review

#### Internet Use and Depressive Symptoms Among Middle-Aged and Older Adults

Numerous studies have examined the relationship between internet use and depressive symptoms in older adults using national representative data sets. Table S1 in Multimedia Appendix 1 summarizes the key findings from these studies. Several insights are worth noting. First, analyses of the correlation between internet use and depressive symptoms yielded inconsistent conclusions. Jun and Kim [27] reported in 2015 that internet use was associated with lower levels of depressive symptoms among Korean older adults. Lee et al [28] showed that older adult cancer survivors in the United States used the internet to complete personal tasks or handle health-related matters, but internet use was unrelated to their depressive symptoms. Lifshitz et al [17] found that the use frequencies of 4 web-based functions (interpersonal communication, information seeking, task performance, and leisure) were not associated with depressive symptoms among Israeli older adults.

Second, some studies have focused on the potential mechanism underlying the association between internet use and depressive symptoms. Internet use is related to a decrease in depressive symptoms among socially inactive older adults [15], and this association is mediated by social isolation [12] and loneliness [22]. Internet use also reduces the negative effect of disadvantageous socioeconomic status (SES) on depressive symptoms [16,29]. However, most studies have used correlation analyses, and only a few studies have revealed a causal effect of internet use on depressive symptoms.

Cotten et al [30] used the propensity score matching method and the cross-sectional Health and Retirement Survey and found that internet use reduced the probability of a depressed state by about 20%-28% among retired older adults in the United States. Subsequently, Cotten et al [12] used a longitudinal design and lagged variables to handle the causal effect, and found that internet use reduced the probability of a depressed state by about 33% among retired older adults in the United States. Nie et al [31] used the IV approach and 2010 cross-sectional China Family Panel Studies (CFPS) data and found that internet use was associated with higher levels of depressive symptoms among Chinese individuals aged 16-60 years. Xie et al [32] also used the CHARLS and propensity score matching method and reported that internet use increased depressive symptoms in Chinese older adults.

This study also uses a nationally representative data set, CHARLS, to examine the effect of internet use on depressive symptoms. We extend the concept of IT use by focusing on the second-level digital divide, which emphasizes the critical role of internet skills. We also attempt to identify causal effects by applying an IV approach to quantify the impact of internet skills on depressive symptoms. In the next section, we elaborate on the second-level digital divide in internet use among older adults and ways to address the endogeneity of internet skills.

#### Internet Skills

The digital divide refers to certain groups having better opportunities than others to benefit from IT. Prior studies have suggested three stages of the digital divide [18,20,26]: (1) economic divide, which implies that some people cannot afford access to IT; (2) usability divide, which emphasizes that IT remains so complicated that some people cannot use it even if they can afford it; and (3) empowerment divide, which refers to inequality of outcomes after IT use. As internet use becomes prevalent among older adults, it is increasingly important to look at who uses the internet and distinguish their internet skills [18,33]. For depression among middle-aged and older adults, the existing literature has primarily discussed the first-level digital divide, which refers to the impact of internet use on depressive symptoms. We further examine the second-level digital divide, namely how internet skills influence depressive symptoms among middle-aged and older adults.

#### IV of Internet Skills

On examining how internet skills affect depressive symptoms, endogenous issues may be raised for two main reasons: (1) internet skills and depressive symptoms are simultaneously affected by unobserved factors such as personality traits, and (2) mentally healthier individuals are more prone to use the internet and have better internet skills. We have used the IV approach to handle potential endogeneity and obtain reliable causal effects.

A few studies have applied the IV approach to capture the effect of internet use. Hong and Chang [34] used the geographical distance to the nearest telecommunication station for each household as an instrumental variable to estimate the impact of internet use on household income at forestry farms in Fujian Province in China. Gao et al [35] applied provincial internet penetration rates as an instrumental variable to capture the effect of computer penetration on Chinese rural farmers’ income. Nie et al [31] used the number of provincial internet broadband access terminals as an instrumental variable to examine the relationship between internet use and depressive symptoms among 16- to 60-year-old Chinese individuals.

This study adopts the above-described “resources accessibility” approach by using the following 2 IV: mobile phone penetration and the performance of government websites. Specifically, higher mobile phone penetration in a city implies that residents have easier access to IT resources, which implies a higher level of IT acceptance and internet skills [31,35]. Similarly, better performance of government website operations indicates higher informatization of the city, which implies higher internet skills of residents in that city [34]. Furthermore, there is no evidence that city-level mobile phone penetration or government website performance is directly associated with individual depressive symptoms. Overall, mobile phone penetration and government website performance correlate with each resident’s internet acceptance and skills, while not being directly correlated with individual depressive symptoms. Therefore, these variables satisfy the principle of IV selection logically [24,36]. Furthermore, when considering the population of older adults in each city, both variables are multiplied by the proportion of the city’s population aged 60 years and older.

## Methods

### Sample and Data Collection

We analyzed data from CHARLS, a nationwide survey designed to provide comprehensive and high-quality data on the demographics, household characteristics, health status and functioning, work, and retirement information of Chinese residents aged 45 years and older [37]. CHARLS is a longitudinal study that used a 4-stage, stratified, cluster sampling design to enroll community-dwelling residents from 450 villages and 150 counties in 28 provinces in China. The national baseline study (wave 1) was conducted in 2011. The last public survey (wave 4) was conducted in 2018, with information obtained from 19,816 respondents.

Since IV regression can investigate the causal effects of independent variables on dependent variables in cross-sectional data, we selected 16,949 participants from CHARLS wave 4. The sampling process is as follows: (1) respondents aged 45 years and older (excluded 178 samples); (2) provided information on internet skills and depression (excluded 2689 samples).

The 2018 Yearbook of China Communication and the 2017 China Government Website Performance Evaluation database provide information on IV. The Yearbook of China Communication provides annual updates on the development of the Chinese telecommunication industry by city. The China Government Website Performance Evaluation contains performance evaluations of government websites for all cities in China, with the latest version being publicly available in 2017.

### Variables

#### Depression

The 10-item Center for Epidemiologic Studies Depression Scale (CES-D-10) is used to examine depressive symptoms. The respondents were asked about their positive feelings, negative emotions, and somatic symptoms during the past week. Scores for each question ranged from 0 to 30, with high scores indicating severe depressive symptoms. Our study considers 12 as a cutoff point to describe the prevalence of depression [38], using the CES-D-10 score in IV regression.

#### Internet Skills

New questions about internet skills have been added in wave 4 of CHARLS. Respondents were asked whether they would use the following web-based functions on their mobile phones: (1) chat on social media (such as WeChat); (2) post on social media (such as WeChat moments); and (3) mobile payments (such as Alipay or WeChat). Respondents provided binary responses, denoting “yes” or “no.” Subsequently, based on the perceived complexity associated with these distinct functions, this study assigned numerical scores to gauge respondents’ internet skills. Respondents capable of using mobile payment systems were assigned a score of 9, those adept at posting on social media received a score of 5, individuals proficient in social media chat were assigned a score of 1, while those abstaining from all the aforementioned functionalities received a score of 0. The cumulative internet skills score was derived by evaluating respondents’ competencies across various functions, within a scale spanning from 0 to 15 points. Higher scores herein signify enhanced internet aptitude.

To reinforce the dependability of our findings, we incorporated an additional measurement strategy, using both a Likert scale with scores ranging from 1 to 5 and a cumulative scoring method as alternative approaches for assessing internet skills. An IV analysis was conducted using these supplementary metrics of internet skills. The inferences drawn from this analysis are in concordance with the foundational model’s findings. Detailed information on the measurements and the corresponding results can be found in Tables S1-S4 in Multimedia Appendix 1.

#### Mobile Phone Penetration and Performance of Government Websites

The first instrumental variable is mobile phone penetration: the number of mobile phone subscribers at the end of the year in a city multiplied by the proportion of individuals aged 60 years and older in each city. Another instrumental variable is the performance of government websites: the score is the sum of the operational scores of each city’s government website, ranging from 50 to 100.

#### Potential Confounding Variables

Potential confounding variables are shown in Table 1.

**Table 1.** Definition/codes of the potential confounding variables.

Variable	Codes/definition
Age	Continuous variable
Gender	0=male; 1=female
Marital status	0=single (divorced, widowed, or single); 1=partnered (married or partnered)
Retirement	0=no; 1=yes
Education	0=less than lower secondary (no formal education, did not finish primary school but can read, private tutoring, elementary school, or middle school); 1=upper secondary (high school or vocational school); 2=tertiary (2- or 3-year college, college graduate, or postgraduate degree)
Total household per capita consumption	Total household consumption or number of people living in the household (skewed distribution, logarithmically transformed)
Residency	0=urban; 1=rural
Ever had a memory problem	Ever had doctor-diagnosed memory-related diseases, including dementia, brain atrophy, and Parkinson disease (0=no; 1=yes)
Ever had a psychological problem	Ever had doctor-diagnosed psychiatric problems, such as emotional, nervous, or psychiatric problems (0=no; 1=yes)
Mobility	A 9-item summary of any difficulty with mobility activities. The mobility activities are walking 100 m, climbing several flights of stairs, getting up from a chair, stooping or kneeling or crouching, extending arms up, lifting 5 kg, and picking up a small coin. Continuous variable: 0-27 (skewed distribution, logarithmically transformed)

### Ethical Considerations

This investigation constitutes a secondary analysis of publicly available data sets; hence, prior registration was not deemed necessary. The foundational data used herein are obtainable via the official portal of the CFPS [39]. Ethical approval for the CFPS was granted from the Biomedical Ethics Committee at Peking University (IRB00001052-14010).

### Statistical Analysis

To examine the effect of internet skills on depressive symptoms, we performed an IV regression to control for possible endogeneity issues. Given that both the dependent and independent variables have skewed distributions, it is meaningful to understand the impact of internet skills on depressive symptoms in terms of percentage change. Thus, we estimate a log-log specification presented in the two-stage least squares model as follows:


(1) $$\log\left(1+cesd_{i}\right)=\beta_{0}+\beta_{1}\log\;\left(1+\hat{internet}\;skill_{i}\right)+\beta_{2}control_{i}+u_{i}$$

(2) $$\log\;\left(1+\hat{internet}\;skill_{i}\right)=\pi_{0}+\pi_{1}\log\;\left(1+mpp_{j}\right)+\pi_{2}control_{i}+v_{ij}$$

where ${cesd}_{i}$ refers to CES-D-10 scores for individual i, ${Internetskill}_{i}$ denotes internet skill scores for individual i, ${control}_{i}$ indicates potential confounding variables, ${mpp}_{j}$ is the mobile phone penetration in city j, and $u_{i},v_{ij}$ are error terms.

We carried out regression analysis to evaluate the association of individual depressive symptoms with individuals’ internet skills and the mobile phone penetration in the city. For another instrumental variable, the first stage of the two-stage least squares model also can be written as follows:


(3) $$\log\;\left(1+\hat{internet}\;skill_{i}\right)=\pi_{0}^{\prime }+\pi_{1}^{\prime }\log\;\left(1+pgw_{j}\right)+\pi_{2}^{\prime }control_{i}+v_{ij}$$

where ${pgw}_{j}$ is the government website performance of city j.

Diagnostic tests including correlation, overidentification, and weak IV tests supported the validity of the aforementioned IV. The data were analyzed using R package AER (version 1.2; R Foundation for Statistical Computing).

## Results

### Descriptive Statistics

The demographic characteristics of the sample are shown in Table 2. There are 16,949 participants, and the mean age in wave 4 (2018) was 62.3 (SD 9.9) years. Most participants are female (n=8735, 51.5%), have a partner (n=14,672, 86.6%), have less than a lower secondary level of education (n=14,619, 86.2%), are currently employed (n=11,086, 66.5%), and are rural residents (n=10,095, 60.7%). In addition, internet skills have significant differences across demographics, including age, gender, and education levels.

**Table 2.** Characteristics of the selected respondents. The total percentage may not equal to 100 due to rounding.

Characteristics		Full sample (n=16,949)	Scores for IT skills					*P* value
			0 (n=14,601, 86.1%)	1 (n=312, 1.8%)	6 (n=558, 3.3%)	10 (n=272, 1.6%)	15 (n=1206, 7.1%)	
Age (years), mean (SD)		62.3 (9.9)	63.3 (9.8)	57.4 (7.7)	59.1 (8.2)	55.1 (7.1)	54.7 (6.4)	<.001^a^
**Gender, n (%)**								.001^b^
	Male	8214 (48.5)	6944 (47.6)	138 (44.2)	294 (52.7)	169 (62.1)	669 (55.5)	
	Female	8735 (51.5)	7657 (52.4)	174 (55.8)	264 (47.3)	103 (37.9)	537 (44.5)	
**Marital status, n (%)**								<.001^b^
	Single	2277 (13.4)	2114 (14.5)	23 (7.4)	42 (7.5)	21 (7.7)	77 (6.4)	
	Partnered	14,672 (86.6)	12,487 (85.5)	289 (92.6)	516 (92.5)	251 (92.3)	1129 (93.6)	
**Education, n (%)**								<.001^b^
	Less than lower secondary	14,619 (86.2)	13,204 (90.4)	241 (77.2)	383 (68.6)	154 (56.6)	637 (52.8)	
	Upper secondary	1939 (11.4)	1242 (8.5)	59 (18.9)	155 (27.8)	81 (29.8)	402 (33.3)	
	Tertiary	391 (2.3)	155 (1.1)	12 (3.8)	20 (3.6)	37 (13.6)	167 (13.9)	
Total household per capita consumption^c^, median		6240.8	5657	7400.2	10,547.5	11,580	13,780	<.001^a^
**Retirement status** ^d^ **, n (%)**								<.001^b^
	Not retired	11,086 (66.5)	9527 (66.1)	208 (68.2)	327 (60.4)	184 (70.5)	840 (73.0)	
	Retired	5575 (33.5)	4877 (33.9)	97 (31.8)	214 (39.6)	77 (29.5)	310 (27.0)	
**Residential area** ^e^ **, n (%)**								<.001^b^
	Urban	6530 (39.3)	5149 (35.8)	146 (48.2)	299 (55.3)	175 (67.6)	761 (66.6)	
	Rural	10,095 (60.7)	9231 (64.2)	157 (51.8)	242 (44.7)	84 (32.4)	381 (33.4)	
**Ever had memory problems** ^f^								.001^b^
	No	16,283 (97.9)	13,977 (97.8)	305 (99.0)	542 (97.7)	270 (99.6)	1189 (99.2)	
	Yes	346 (2.1)	320 (2.2)	3 (1.0)	13 (2.3)	1 (0.4)	9 (0.8)	
**Ever had psychological problems** ^g^								.25^b^
	No	16,512 (98.9)	14,197 (98.9)	309 (99.7)	548 (99.1)	268 (99.3)	1190 (99.4)	
	Yes	179 (1.1)	164 (1.1)	1 (0.3)	5 (0.9)	2 (0.7)	7 (0.6)	
Mobility^h^, mean (SD)		4.1 (5.1)	4.4 (5.3)	2.7 (3.7)	2.2 (3.0)	1.5 (2.6)	1.1 (2.0)	<.001^a^
CES-D-10^i^ score, mean (SD)		8.4 (6.5)	8.8 (6.6)	7.2 (6.0)	6.7 (5.5)	6.1 (5.3)	6.1 (5.3)	<.001^a^
Mobile phone user rate, mean (SD)		127.7 (127)	125.6 (125)	131 (133.6)	139.5 (148.7)	143 (132.8)	142.3 (136)	<.001^a^
Government website performance, mean (SD)		63.8 (15.1)	63.5 (14.9)	64.2 (15.4)	64.4 (15.9)	66.4 (15.7)	66.8 (15.9)	<.001^a^

a Outcome of the Kruskal-Wallis test.

b Outcomes of the chi-square test.

c Missing data: n=2459.

d Missing data: n=288.

e Missing data: n=324.

f Missing data: n=320.

g Missing data: n=258.

h Missing data: n=44.

i CES-D-10: 10-item Center for Epidemiologic Studies Depression Scale.

Table 1 reveals a distinct profile among individuals with elevated internet skill scores. These individuals are predominantly younger, hail from higher-income households (higher total household per capita consumption), are more likely to be male, have partners, are nonretired, and are predominantly urban residents. Additionally, this cohort is characterized by superior health outcomes, evident from lower incidences of memory-related issues, psychological problems, and reduced mobility challenges.

Further analysis of internet skill scores underscores a stark digital divide. A significant 86.1% of participants do not engage with the internet, thereby receiving a score of 0. Conversely, within the subset of internet users, those with advanced internet skills form the majority, representing 7.1% of the overall sample and 51.4% of internet users (1206 out of 2348 individuals). This disparity highlights not only a first-level digital divide but also a pronounced second-level digital divide within the middle-aged and older demographic, marked by a dual extremity of complete absence or high proficiency in internet skills.

### Prevalence of Depression in Residents

The prevalence of depression varied among participants with different levels of internet skills. Respondents with lower-level internet skills are at higher risk of depression than those with higher-level internet skills (30.23% vs 20.51% vs 17.38% vs 13.24% vs 15.17%).

### The Effect of Internet Skills on Depressive Symptoms in Residents

The association between internet skills and depressive symptoms was estimated using ordinary least squares (OLS) regression, and the effect of internet skills on depressive symptoms was subsequently estimated using IV regression (see Table 3). OLS regression suggests that a 1% increase in internet skills is associated with a 0.037% decrease in depressive symptoms (β=–.037, SE 0.009), which underestimates the causal effect. As expected, internet skills are an endogenous variable (*F* test *P* value <.001). IV regressions indicate that a 1% increase in internet skills leads to a 1.135% (SE 0.471) to 1.741% (SE 0.297) reduction in depressive symptoms. The 2 IV were neither weak (*F*_–1_=16.7 and 28.5 both being >10) nor endogenous (Wu-Hausman test *P* value of .10; being >.05 or .01). Detailed results of the controls can be found in Table S5 in Multimedia Appendix 1.

**Table 3.** The results of ordinary least squares (OLS) and instrumental variables (IV) regression analyses. The potential confounding variables are controlled in all models.

		OLS	IV	
			Mobile phone penetration	Performance of government websites
Log (internet skills), β (SE)		–0.037 (0.009)^a^	–1.741 (0.471)^a^	–1.135 (0.297)^a^
Demographic variables		✓^b^	✓	✓
Health status		✓	✓	✓
Constant, β (SE)		2.236 (0.068)^a^	4.015 (0.503)^a^	3.383 (0.322)^a^
Observations, n		16,949	16,949	16,949
**Correlation with internet skills (first stage regression)**				
	α (SE)	—^c^	0.032 (0.008)^a^	0.136 (0.026)^a^
	Weak IV (*F* test)	—	Not supported	Not supported
	*F* test (*df*)	—	16.7 (–1)^a^	28.5 (–1)^a^
	Exogenous to depression (Wu-Hausman test)	—	IV is exogenous	IV is exogenous
	*P* value	—	.10	.10
	Endogenous to depression (*F* test)	—	Internet skills is endogenous	Internet skills is endogenous
	*P* value	—	<.001	<.001

a *P*<.001.

b Demographic variables and health status have been controlled for in the model. To maintain the conciseness of the model’s results, the coefficient estimates for demographic and health status variables are shown in Table S6 in Multimedia Appendix 1 rather than in the main text.

c Not available.

### Sensitivity Analysis

We applied multiple imputation techniques to missing values, assuming that missing variables are missing at random. To examine the robustness of the results, we conducted OLS and IV regressions with missing values and compared the results. Our findings yielded no difference between complete cases and the prior sample. Detailed results can be found in Table S6 in Multimedia Appendix 1.

## Discussion

This study explored the impact of internet skills on depressive symptoms. Our findings revealed that (1) internet skills are relatively low among Chinese middle-aged and older adults and (2) improvement of internet skills can reduce depressive symptoms.

### Impact of Internet Skills on Depressive Symptoms

Our results suggest that for middle-aged and older Chinese adults, a 1% increase in their internet skills leads to a 1.1% to 1.7% reduction in depressive symptoms. Our main finding is consistent with those of Cotten et al [12,30], who reported a positive outcome of internet use reducing risk of depression by 20% to 33% among retired older adults in the United States. We used internet skills (0~15 points) to measure IT use rather than using dummy variables. In addition, since the CES-D-10 scores are a continuous variable, we could estimate the effect of internet skills on depressive symptoms.

Some studies have reported that internet use increases depressive symptoms in the context of China [31,32]. Nie et al [31] used the IV approach and 2010 CFPS data and found that internet use was associated with higher levels of depressive symptoms among Chinese individuals aged 16-60 years. Notably, in 2010, internet access among middle-aged and older Chinese individuals was limited [40], and some residents were even prejudiced and resistant to the internet [31]. These reasons may lead to a negative impact of internet use on depression. The existing literature predominantly addresses the first-level digital divide, examining the effect of internet use on depressive symptoms [31,32]. Our study expands on this by delving into the second-level digital divide, revealing that enhanced internet skills significantly mitigate depressive symptoms. This observation, divergent from prior studies, suggests a possible shift in the perception of the internet among the middle-aged and older Chinese individuals as it becomes more ingrained in various facets of society [31]. Proficiency in internet use potentially enables more effective usage [13,16,19], leading to positive psychosocial outcomes. For instance, improved internet skills facilitate web-based social interactions and content sharing, thus increasing social support and reducing isolation and loneliness [11,15,22,27]. Further skill enhancement allows these individuals to more efficiently perform daily activities such as shopping and bill payments on the web, fostering a greater sense of inclusion and autonomy in the digital era [14,16,19]. Such psychosocial benefits potentially alleviate depressive symptoms and bolster mental health.

Our study additionally revealed that populations with a higher SES and superior health tend to exhibit more advanced internet skills. While it has been acknowledged that SES can create disparities in resource access and health outcomes [29,33], our research indicates that the improvement of internet skills might mitigate depressive symptoms, even in scenarios where SES and health factors are consistent. This finding suggests that diminishing the second-level digital divide could play a role in lessening health disparities. Consequently, future research should delve into the potential mechanisms by which internet skills influence depressive symptoms and examine the interplay between this subject and health equity concerns.

### Improving Internet Skills Among Middle-Aged and Older Residents

This study delves into the usage of the internet among middle-aged and older adults in China, analyzing it through the digital divide framework. As of 2018, around 86% of respondents either demonstrated limited internet skills or lacked access altogether. This figure represents an improvement from the 98% and 97% non–internet use rate observed between 2011 and 2015 [38], indicating a gradual increase in both internet usage and skill acquisition among this demographic. Such a trend suggests an ongoing closure of the first-level digital divide, marked by a shift from nonuse to initial internet engagement. Despite this progress, a second-level digital divide is apparent among those who have embraced the internet and acquired specific skills, with a substantial segment showing advanced internet skills, underscoring an uneven skill distribution. The variability in internet skills may be attributed to individual IT preferences and the consistent support from family and peers in IT usage and learning [20,31,41]. Considering the beneficial impact of internet skills on the mental health of middle-aged and older individuals, and in light of increasing internet penetration rates, it becomes crucial for society and policy makers alike to focus on bridging this second-level digital divide.

IT training programs can reduce anxiety in older adults, increase their interest and efficacy in IT, and improve their IT capability [42]. A community or senior university that provides courses on internet use can follow three guidelines: (1) provide regular long-term training: older adults prefer long-term guidance on IT use and a stable context for experience exchange [42]; (2) leverage the influence of those with good internet skills [43]: interacting with more qualified peers and obtaining guidance from them will reduce the attrition rate of courses; more skilled older adults are important role models and mentors for older adults to improve their skills [43]; and (3) develop web-based participation projects to encourage older adults to establish, maintain, and participate in their own web-based communities [44].

Technical support from the younger generation is vital: young people are “impatient” with older IT learners and lack an understanding of older adults’ special needs [41]. We need to educate younger generations about the benefits of internet use among older adults to inspire families and communities to bridge the digital divide for older adults. Improving internet skills also requires gerontological software design improvements. The Chinese government is actively promoting the gerontological design of internet-based applications [45]. The initial stages of the project mainly focus on interface design and function simplification. Follow-up studies should develop tools and applications that specifically support older adults’ current activities and goals. Finally, older adults may have negative attitudes toward the internet [31]. It is necessary to reduce the negative connotations surrounding internet use and emphasize that web-based activity can be meaningful and manageable rather than just a form of entertainment.

### Limitations

This study has several limitations. First, our findings are based on self-reported data, which implies potential self-reported bias. Second, we assume that the data are missing at random and used multiple imputations to resolve this issue. However, the excluded individuals are more likely to be older, less educated, have a severe disability, and more likely to have higher levels of depressive symptoms. Thus, we cannot exclude this bias. Third, while CHARLS provides a substantial sample size for investigating the relationship between internet skills and depressive symptoms, it is imperative to acknowledge certain shortcomings inherent in the measurement items within the secondary data. This study delineates and quantifies internet skills, differentiated by the level of difficulty associated with various functionalities. Future research should aim to explore more robust measurement methodologies to enhance the reliability of these findings. Lastly, in 2018, a significant majority (98.6%) of Chinese internet users accessed the internet via their mobile phones [46]. Consequently, this study used the rate of mobile phone penetration at the city level as an instrumental variable. This approach was further nuanced by incorporating the percentage of residents aged 60 years and older in each city. Future research should aim to enhance the precision of this instrumental variable by integrating data on city-level internet usage. Additionally, examining the segment of the population aged 45 years and older may offer more relevance, closely mirroring the age range of our study’s sample. Future research can address these issues and explore the formative mechanisms of the second-level digital divide among older adults.

### Conclusion

Understanding the role of IT in fulfilling the well-being of older adults has been limited. This study evaluates the effect of internet skills on depressive symptoms through the IV approach. The results reveal the emergence of a second-level digital divide—the disparity in internet skills among middle-aged and older Chinese adults. A 1% improvement in internet skills reduces depressive symptoms by 1.1%-1.7%. This study contributes to the literature on the societal impacts of the internet.

## Supplementary material

10.2196/50880 Multimedia Appendix 1 Key findings from the literature review, results from the analysis of alternative approaches for assessing internet skills, detailed results of the controls, and detailed results of the ordinary least squares and instrumental variables regressions.

## Abbreviations

CES-D-10: 10-item Center for Epidemiologic Studies Depression Scale
CFPS: China Family Panel Studies
CHARLS: China Health and Retirement Longitudinal Study
IV: instrumental variables
OLS: ordinary least squares
SES: socioeconomic status
