# Supplementary material for: Assessing the Impact of Internet Skills on Depressive Symptoms Among Chinese Middle-Aged and Older Adults: Cross-Sectional Instrumental Variables Analysis
Source: JMIR Aging. 2024 Mar 21;7:e50880. doi: 10.2196/50880 (PMC11004627; doi:10.2196/50880)
Supplement: Multimedia Appendix 1 [file aging-v7-e50880-s001.docx]

Table S1. Review of studies on the relationship between Internet use and depression.

| Author (year), location of the study site | Study design, sample size | IT measures | Depressive symptoms measures | Outcome | Limitation |
| --- | --- | --- | --- | --- | --- |
| Elliot et al., (2014), USA | A cross-sectional study, data from the National Health and Aging Trends Study (NHATS) 2011, 6,443 participants (aged ≥ 65) | Use the communications technology (CT: e-mailed or texted) or information technology (computer or Internet) in the past month  CT: 0 = none to 3 = most days  IT: 0 = did not have or use a computer; 1 = had or used a computer but had not gone on the Internet; 2 = had used the Internet but not for both shopping/banking and health-related purposes; 3 = used the Internet for at least one activity in each category | 2-item version of the Patient Health Questionnaire (PHQ-2) | ICT use is unrelated to depressive symptoms. | Restricted to “related” relation.  The concept of Internet use is restricted by a first-level digital divide - "access". |
| Lee et al., (2018), USA | A cross-sectional study, data from the NHATS 2011, 1,411 participants (aged ≥ 65, Cancer survivors) | NHATS IT measures  CT: 0 = none/rarely to 2 = most days  IT: completing any personal tasks (yes/no)  handling any health matters (yes/no) | PHQ-2 | IT use is unrelated to depressive symptoms.  CT use is associated with decreased risk for severe depressive symptoms. | Restricted to “related” relation.  The concept of Internet use is restricted by a first-level digital divide - "access". |
| Kim et al., (2020), USA | A cross-sectional study, data from the NHATS 2011, 4,976 participants (aged ≥ 65) | NHATS IT measures  Regrouped in 4 groups: ICT users, CT only users, IT only users, and nonusers) | PHQ-2 | IT or CT use only is unrelated to depressive symptoms.  ICT users are less likely to experience major depressive symptoms than nonusers. | Restricted to “related” relation.  The concept of Internet use is restricted by a first-level digital divide - "access". |
| Cotten et al., (2012), USA | A cross-sectional study, data from the HRS 2006, 7,839 participants (retired, aged ≥ 50) | Regularly use the Internet (yes/no) | 8-item version of the Center for Epidemiologic Studies scale (CES-D8) | Internet use reduces the probability of a depressed state by about 20-28%. | The concept of Internet use is restricted by a first-level digital divide - "access". |
| Cotten et al., (2014), USA | A Longitudinal study, data from the Health and Retirement Survey (HRS) 4 waves (2002-2008), 3,075 participants (retired, aged ≥ 50) | Regularly use the Internet (yes/no) | CES-D8 | Internet use reduces the probability of a depressed state by about 33%.  Reduction in depression is largest for people living alone. | The concept of Internet use is restricted by a first-level digital divide - "access". |
| Chopik, (2016), USA | A cross-sectional study, data from the HRS 2012, 591 participants (aged ≥ 50) | Use the Internet for 4 types of social functions (counting all types of activities) | CES-D8 | Internet use for social connection is associated with lower levels of depressive symptoms.  This relation is mediated by reduced loneliness. | Restricted to “related” relation.  The concept of Internet use is restricted by a first-level digital divide - "access". |
| Lifshitz et al., (2018), Israel | A cross-sectional study, data from the online survey, 306 participants (aged ≥ 50, Internet users) | The intensity of use the 4 types (communication, information seeking, task performance, and leisure) of online functions in the last week (1 = very rarely to 5 = very frequently) | CES-D11 | All 4 online functions used are unrelated to depressive symptoms. | Restricted to “related” relation.  The concept of Internet use is restricted by a first-level digital divide - "access".  Small sample size. |
| Jun & Kim, (2016), Korea | A longitudinal study, data from the Korean Welfare Panel Study 4 waves (2012-2015), 6,306 participants (aged ≥ 50) | Use the Internet in the last year (yes/no) | CES-D11 | Internet use is associated with lower levels of depressive symptoms. | Restricted to “related” relation.  The concept of Internet use is restricted by a first-level digital divide - "access". |
| Lee et al., (2020), Korea | A cross-sectional study, data from the Survey of Living Conditions and Welfare Needs of Korean Older Persons 2017, 10,055 participants (aged ≥ 65) | Use the Internet for 9 types of online activities (yes/no, counting all types of activities, scores ranged 0-9) | Short-form Korean version of the Geriatric Depression Scale (GDSSF-K) | Internet use is associated with lower levels of depressive symptoms.  This benefit is larger for those who have fewer social interactions with friends or neighbors. | Restricted to “related” relation.  The concept of Internet use is restricted by a first-level digital divide - "access". |
| Nie et al., (2017), China | A cross-sectional study, data from the China Family Panel Studies (CFPS) 2010, 4,686 participants (Internet user, aged 16-60) | The intensity of the Internet use (hours/day) | 6-item depression-related conditions (1 = almost every day to 5 = never, 6-30) | Internet use is associated with higher levels of depressive symptoms. | Restricted to “related” relation.  The concept of Internet use is restricted by a first-level digital divide - "access".  Excessive age range may not capture older adults’ results. |
| Wu & Chiou, (2020), Taiwan, China | A cross-sectional study, data from the survey conducted in 2017, 153 participants (aged ≥ 60) | Familiar with the use of social media (yes/no) | GDS-15 | Familiarity with social media is associated with lower levels of depressive symptoms. | Restricted to “related” relation.  The concept of Internet use is restricted by a first-level digital divide - "access".  Not a nationally representative dataset. |
| Yuan, (2020), Shanghai, China | A cross-sectional study, data from the Shanghai Urban Neighborhood Survey, 2,117 participants (aged 60-80) | Frequency of use the Internet for 4 types of online activities (0 = never to 3 = all the time, summarize all types of activities, scores ranged 0-12) | 10-item of the Hopkins Symptom Checklist | More frequent Internet use is associated with lower odds of having mental health problems.  This benefit is greater for those with chronic diseases and low income. | Restricted to “related” relation.  The concept of Internet use is restricted by a first-level digital divide - "access".  Not a nationally representative dataset. |
| Mu et al., (2021), China | A cross-sectional study, data from the CHARLS 2015, 8,853 participants (aged ≥ 45) | Use the Internet in the past month (yes/no) | CES-D10 | Internet use is associated with lower levels of depressive symptoms.  The negative effect of low levels SES on depressive symptoms is reduced by Internet use. | Restricted to “related” relation.  The concept of Internet use is restricted by a first-level digital divide - "access". |
| Xie et al., (2021), China | A longitudinal study, data from the China Longitudinal Aging Social Survey 2 waves (2014-2016), 6,972 participants (aged ≥ 60) | Regularly use the Internet (yes/no) | CES-D9 | Internet use increases the incidence of depressive symptoms. | The concept of Internet use is restricted by a first-level digital divide - "access". |

Table S2. Alternative measurements for assessing internet skills

| Internet skills | Original measurement | Cumulative scoring | Likert scoring |
| --- | --- | --- | --- |
| Non user | 0 | 0 | 1 |
| Chat on social media | 1 | 1 | 2 |
| Chat on social media + post on social media | 6 | 2 | 3 |
| Post on social media + mobile payment | 10 | 2 | 4 |
| Chat on social media + post on social media + mobile payment | 15 | 3 | 5 |

Table S3. Scores of alternative measurements

| Internet skills | | Mean (SD) | n (%) |
| --- | --- | --- | --- |
| Cumulative scoring |  |  |  |
|  | 0 | 0.3 (0.9) | 14601 (86.1%) |
|  | 1 |  | 312 (1.8%) |
|  | 2 |  | 830 (4.9%) |
|  | 3 |  | 1206 (7.1%) |
| Likert scoring |  |  |  |
|  | 1 | 1.4 (1.1) | 14601 (86.1%) |
|  | 2 |  | 312 (1.8%) |
|  | 3 |  | 558 (3.3%) |
|  | 4 |  | 272 (1.6%) |
|  | 5 |  | 1206 (7.1%) |

Table S4. The results of IV regression using alternative measurements of internet skills

|  | Cumulative scoring | | Likert scoring | |
| --- | --- | --- | --- | --- |
|  | IV: mpp | IV: pgw | IV: mpp | IV: pgw |
|  | β (SE) | β (SE) | β (SE) | β (SE) |
| Log (Internet skills) | -1.342*** | -0.912*** | -3.144*** | -2.046*** |
|  | (0.372) | (0.248) | (0.851) | (0.534) |
| Demographic controls | √ | √ | √ | √ |
| Health status | √ | √ | √ | √ |
| Constant | 1.034*** | 1.407*** | 4.366*** | 3.609*** |
|  | (0.348) | (0.237) | (0.596) | (0.378) |
| Observations | 16,949 | | | |

Notes: *p < 0.05, **p < 0.01, ***p < 0.001

Table S5. The results of OLS and IV regression in complete cases

|  | OLS | IV: mpp | IV: pgw |
| --- | --- | --- | --- |
|  | β (SE) | β (SE) | β (SE) |
| Log (Internet skills) | -0.031*** | -1.637*** | -1.306*** |
|  | (0.009) | (0.487) | (0.381) |
| Demographic controls | √ | √ | √ |
| Health status | √ | √ | √ |
| Constant | 2.261*** | 4.015*** | 3.654*** |
|  | (0.076) | (0.546) | (0.430) |
| Observations | 13,349 | 13,349 | 13,349 |
| Correlation with Internet skills (First stage regression) |  |  |  |
| α (SE) |  | 0.034^***^ | 0.129^***^ |
|  |  | (0.009) | (0.030) |
| Weak IV (F test) |  | Not supported | |
| F value |  | 14.1*** | 19.9*** |
| Exogenous to depression (Wu-Hausman test) |  | IV is exogenous | |
| p-value |  | 0.4376 | |
| Endogenous to depression (F test) |  | IT skill is endogenous | |
| p-value |  | <0.001 | <0.001 |

Notes: The potential confounding variables are controlled in all models; *p < 0.05, **p < 0.01, ***p < 0.001

Table S6. The results of OLS and IV regression

|  | OLS | IV: mpp | IV: pgw |
| --- | --- | --- | --- |
|  | β (SE) | β (SE) | β (SE) |
| Log (Internet skills) | -0.037*** | -1.741*** | -1.135*** |
|  | (0.009) | (0.471) | (0.297) |
| Demographic controls |  |  |  |
| Age | -0.008*** | -0.037*** | -0.027*** |
|  | (0.001) | (0.008) | (0.005) |
| Gender (reference is male) |  |  |  |
| female | 0.099*** | 0.039 | 0.061*** |
|  | (0.013) | (0.029) | (0.021) |
| Marital status (reference is single) |  |  |  |
| partnered | -0.134*** | -0.311*** | -0.248*** |
|  | (0.019) | (0.058) | (0.040) |
| Education (reference is less than lower secondary) | |  |  |
| upper secondary | -0.107*** | 0.716*** | 0.424*** |
|  | (0.021) | (0.234) | (0.149) |
| tertiary | -0.161*** | 1.651*** | 1.008*** |
|  | (0.046) | (0.518) | (0.329) |
| Log (total household per capita consumption) | -0.008* | 0.094*** | 0.058*** |
|  | (0.005) | (0.029) | (0.019) |
| Retirement (reference is not retired) |  |  |  |
| retired | -0.077*** | -0.044 | -0.056*** |
|  | (0.014) | (0.028) | (0.021) |
| Residency (reference is urban) |  |  |  |
| rural | 0.122*** | -0.181** | -0.074 |
|  | (0.014) | (0.087) | (0.056) |
| Ever had memory problems (reference is no) |  |  |  |
| yes | 0.196*** | 0.222*** | 0.213*** |
|  | (0.043) | (0.069) | (0.055) |
| Ever had psych problems (reference is no) |  |  |  |
| yes | 0.147** | 0.112 | 0.124* |
|  | (0.059) | (0.095) | (0.076) |
| Log (mobility) | 0.287*** | 0.161*** | 0.206*** |
|  | (0.007) | (0.037) | (0.024) |
| Constant | 2.236*** | 4.015*** | 3.383*** |
|  | (0.068) | (0.503) | (0.322) |
| Observations | 16,949 | 16,949 | 16,949 |
